# Percutaneous Closure of a Large Right Coronary Artery Fistula Draining Into the Superior Vena Cava

**DOI:** 10.1016/j.jaccas.2025.105013

**Published:** 2025-09-17

**Authors:** Mickael Hermano Ogama, Pablo Tomé Teixeirense, Marden A. Tebet, Vinícius Esteves, João Ricardo Antunes, Ariane Binoti Pacheco, Igor Ramon Batista, Sérgio Kreimer, Eduardo Moreira

**Affiliations:** a Hospital São Luiz Jabaquara, São Paulo, Brazil; b Hospital Fornecedores de Cana, Piracicaba, Brazil; c MultiScan Diagnostics, Vitoria, Brazil

**Keywords:** cardiac catheterization, congenital heart disease, coronary fistula

## Abstract

**Background:**

Coronary fistulas exhibit considerable anatomic diversity, resulting in unique implications and treatment approaches.

**Case Summary:**

A 38-year-old man with palpitations, chest pain, and dizziness was diagnosed with a large coronary fistula connecting the proximal right coronary artery to the superior vena cava. Percutaneous occlusion was chosen to alleviate symptoms and elevated right-sided filling pressures. After angiotomographic and angiographic evaluation, vascular plugs were selected because of the large caliber of the fistula.

**Discussion:**

This case highlights that although rare, coronary fistulas can cause significant clinical repercussions, and their management must be based on a comprehensive assessment of both clinical and anatomic factors.

**Take-Home Messages:**

Coronary fistulas can cause significant symptoms and clinical repercussions, which warrant an evaluation for occlusion. The management of coronary fistulas requires a multidisciplinary heart team discussion to select the optimal treatment based on precise clinical and anatomic considerations.

## Graphical Abstract

**Figure:**
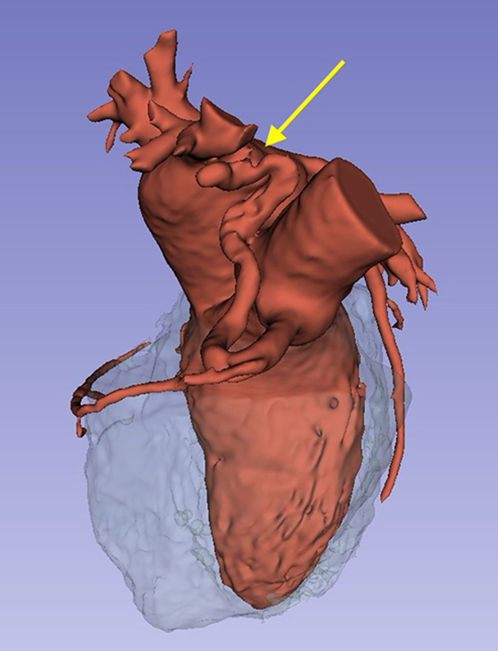

## History of Presentation

A 38-year-old man presented to the emergency department with complaints of palpitations accompanied by chest pain and dizziness, which began abruptly. Physical examination revealed no abnormalities on admission. An electrocardiogram revealed sinus rhythm and an early repolarization pattern.

**Take-Home Messages**

• Coronary fistulas can cause symptoms and repercussions and require specific treatment.
• Given its heterogeneity and the limited number of studies, the selection of the optimal treatment modality, whether surgical or percutaneous—using coils, plugs, or covered stents, and administered via arterial, venous, or postarteriovenous connection—must be determined based on clinical and anatomic considerations after a multidisciplinary discussion.

## Past Medical History

He reported a history of former smoking (15 years at 1 pack per day).

## Differential Diagnosis

The patient was subsequently admitted for evaluation, with the considerations of acute coronary syndrome and tachyarrhythmia being proposed.

## Investigations

**Figure 1:**
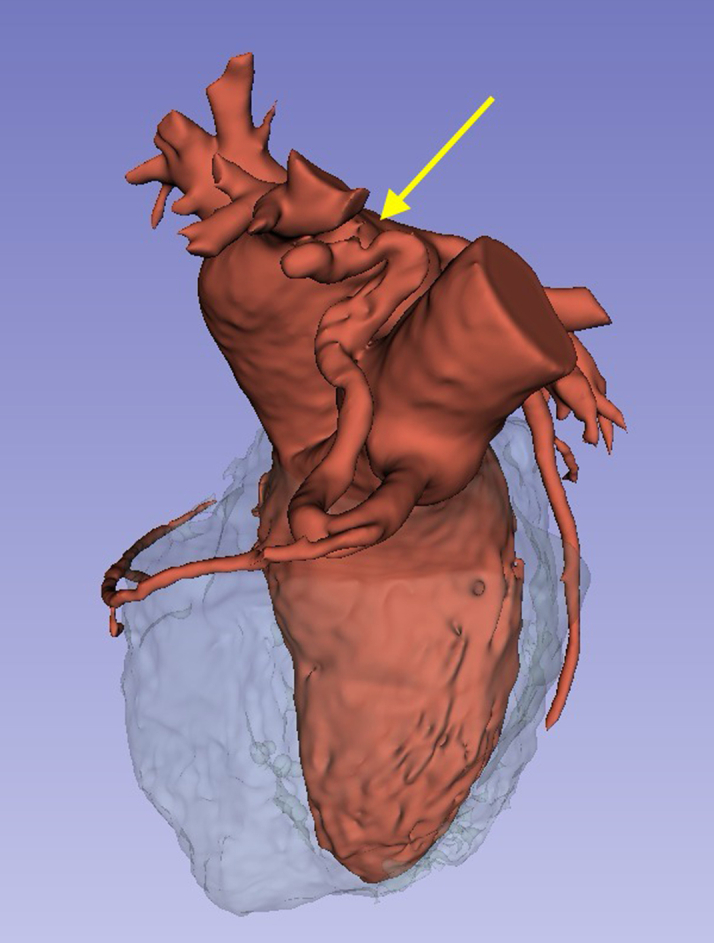
3-Dimensional Reconstruction of the Angiotomography Using the Open Software 3D Slicer The tortuosity of the fistula originating from the proximal third of the right coronary artery and terminating in the superior vena cava is observable (yellow arrow).

Troponin levels were assessed and found to be negative, and an echocardiogram revealed no abnormalities. Coronary angiotomography revealed a coronary calcium score of zero and the absence of stenoses; however, a fistula was identified between the right coronary artery (a branch of the sinus node) and the superior vena cava (Figure 1). Holter monitoring was conducted, revealing 8% of ventricular extrasystoles over a 24-hour period. After the results of the coronary angiotomography, the patient was referred for cardiac catheterization, which verified the absence of coronary stenoses and identified a significant fistula between the proximal third of the right coronary artery and the superior vena cava. Hemodynamic study indicated mild precapillary pulmonary hypertension (right ventricular pressure: 35/3/14 mm Hg, pulmonary pressure: 35/15/21 mm Hg) accompanied by elevated filling pressures in the right chambers. Oximetric evaluation showed a step up at the superior vena cava level, with a Q_p_/Q_s_ of 1.5.

## Management (Medical/Interventions)

**Figure 2:**
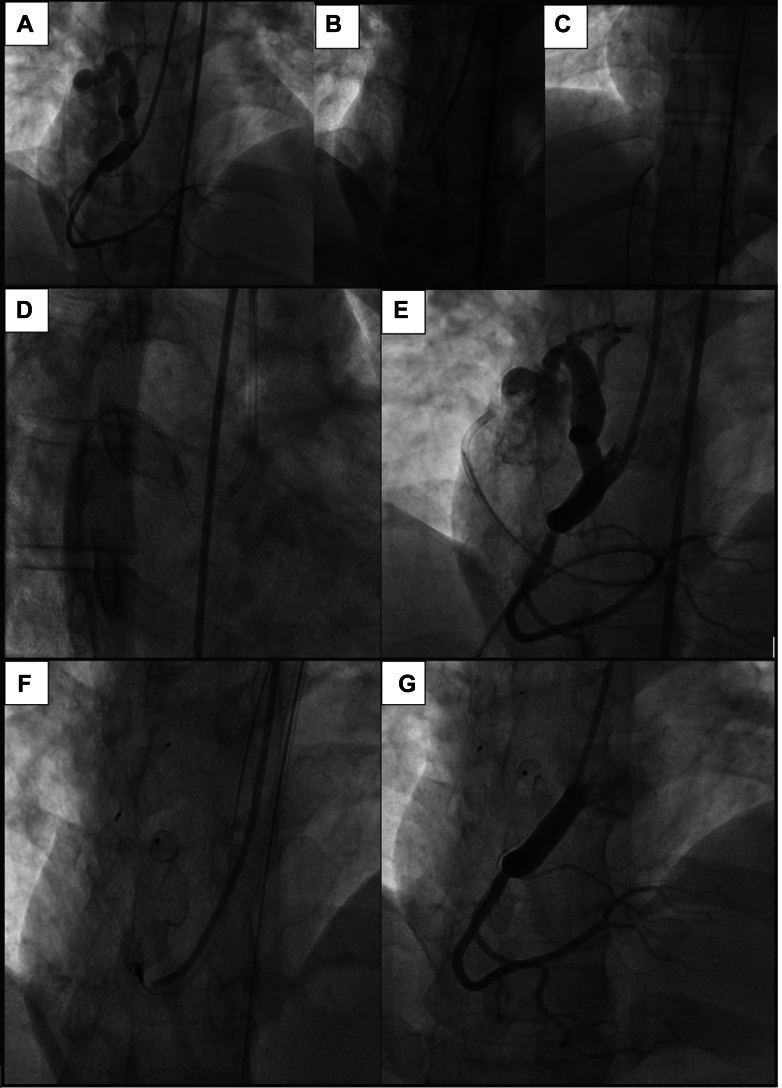
Percutaneous Occlusion Through Plugs in the Arterial and Venous Ends of the Coronary Fistula (A) Initial injection demonstrating a large fistula between the proximal third of the right coronary artery and the superior vena cava. (B) Passage of a 0.014-in × 300-cm guidewire through the fistula with the aid of a microcatheter. (C) Formation of an arteriovenous loop with the aid of a snare. (D) Advancement of the guide catheter through the venous route with the aid of balloon-assisted tracking. (E) An 8-mm AVP 4 vascular plug released at the venous end and a (F) 12-mm AVP II vascular plug released at the arterial end. (G) The final injection demonstrates no residual flow through the fistula.

The findings prompted the initiation of a beta-blocker for the elevated frequency of ventricular extrasystoles and a statin for previously untreated hyperlipidemia. The fistula was occluded percutaneously because of its substantial size, which was associated with elevated filling pressures in the right chambers and the potential for symptomatic manifestations or arrhythmias. The procedure was performed through right femoral venous and arterial access (8-F) and right radial arterial access (6-F). The right coronary artery was cannulated with a JR 7-F guiding catheter. Coronary angiography disclosed a large fistula characterized by an elongated, convoluted trajectory originating from the third proximal segment and extending to the superior vena cava. A microcatheter facilitated the advancement of a 0.014-inch × 300-cm guidewire through the fistula and into the superior vena cava. With a 10-mm snare catheter, the 0.014-inch × 300-cm wire was captured for the arteriovenous rail. It was possible to place a JR 4.0 6-F guiding catheter in the fistula's venous inlet with the help of an arteriovenous track and the balloon-assisted tracking technique with a 2.0- × 15-mm noncompliant balloon. Multiple attempts to release Amplatzer Vascular Plug II 10-mm and Amplatzer Vascular Plug II 8-mm occluders were unsuccessful. Then, we successfully released 1 Amplatzer Vascular Plug 4 8-mm device (Abbott Laboratories) in the venous inlet. With the help of a JR 3.5 5-F diagnostic catheter and a 0.035-inch × 260-cm super stiff guidewire, a 90-cm 6-F sheath was placed in the middle third of the fistula through the arterial inlet. A 12-mm Amplatzer Vascular Plug II occluder was successfully released through the arterial inlet (Figure 2).

## Outcome and Follow-Up

**Figure 3:**
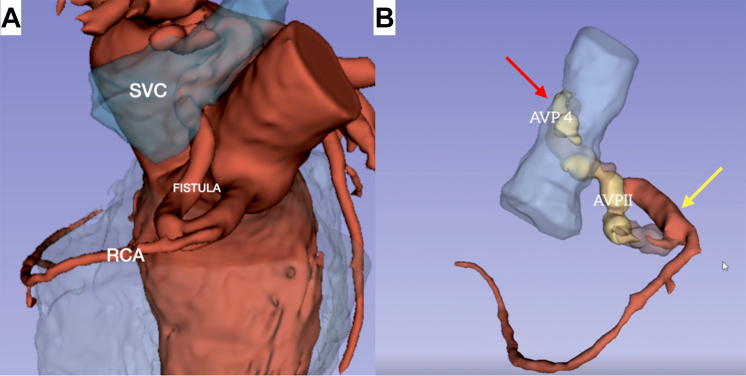
3-Dimensional Reconstruction of the Fistula, Before and After Occlusion (A) Preprocedural reconstruction image illustrating a large fistula between the proximal third of the right coronary artery and the superior vena cava. (B) Postprocedural reconstruction image illustrating a thrombosed fistula, featuring vascular occlusion devices at both the arterial (yellow arrow = RCA) and the venous (red arrow = SVC) termini.

The patient experienced no complications during the postoperative period. The postoperative echocardiogram yielded normal results, and a subsequent coronary angiotomography indicated no residual flow through the fistula. A 3-dimensional reconstruction of the fistula was performed, and it is possible to observe the devices in place, and thrombus formed between them (Figure 3, Video 1). The patient was discharged on the second postoperative day, with a regimen of complete anticoagulation (apixaban) for 1 month, succeeded by dual antiplatelet therapy for 6 months.

## Discussion

The prevalence of coronary fistulas exhibits significant variability across studies and is deemed uncommon (0.1%-0.2% of the population),^1^ notwithstanding a recent rise in diagnosis rates attributed to enhanced imaging techniques. Most are incidental discoveries. They can be categorized based on various anatomic characteristics: vessel of origin, segment of origin (Sakakibara type A vs Sakakibara type B), site of termination (coronary arteriovenous fistula vs coronary cameral fistula), fistula morphology (simple vs complex fistula), number of fistulas (single vs multiple), and angiographic size. Sakakibara type A describes a fistula that originates from the proximal third of the native vessel, whereas Sakakibara type B describes a fistula that forms beyond the proximal third or the continuation of the native vessel. The morphology of fistulas is crucial for their classification. A fistula is deemed simple if it arises from one vessel and has a singular termination. Complex fistulas are distinguished by plexiform variants. Angiography is significant for classifying fistulas by size, but it is limited to simple macrofistulas. A fistula is classified as large if it exceeds twice the diameter of the distal reference vessel, medium if it is 1 to 2 times that diameter, and small if it is less than the diameter of the distal reference vessel.^2^

Clinically, individuals may be asymptomatic or exhibit symptoms resulting from steal of flow and its repercussions (chamber dilation, heart failure, arrhythmias, and ischemia), thromboembolization (acute myocardial infarction), infection, and, less commonly, rupture. Studies are scarce, and management is based mainly on expert opinion derived from case series.^3^^,^^4^ Surgical or percutaneous interventions are presently advised for symptomatic patients with medium-sized fistulas (fistula diameter ranging from 1 to 2 times the largest diameter of the coronary vessel not supplying the fistula) or large-sized fistulas (diameter exceeding 2 times), because of complications such as ischemia, chamber dilation, ventricular dysfunction, arrhythmias, endarteritis, and rupture. The decision between surgical and percutaneous treatment will depend on the viability of percutaneous methods as determined by imaging, the necessity for additional surgical interventions, and the associated risks of each approach.^5^

## Conclusions

Coronary fistulas are infrequent and typically asymptomatic; however, they may be linked to myocardial involvement and symptoms, necessitating specific treatment because of their prognostic significance. Advancements in percutaneous treatment modalities occur as new materials are developed and expertise is gained. Further research is required to elucidate indications, methodologies, recurrence rates, and prognostic outcomes.

**Visual Summary:** Patient's Treatment Timeline

Timeline	Events
Admission to the ED	A 38-year-old man was admitted to the ED with complaints of palpitations, chest pain, and dizziness. Physical examination was unremarkable, and the electrocardiogram showed only an early repolarization pattern. Myocardial necrosis markers were negative, and echocardiogram was normal.
Day 1	Coronary angiotomography is performed, which does not visualize coronary stenosis but identifies a fistula between the proximal third of the right coronary artery and the superior vena cava.
Day 2	Cardiac catheterization is conducted, confirming the absence of coronary stenosis and the presence of a large fistula between the proximal third of the right coronary artery and the superior vena cava. Manometry shows mild pulmonary hypertension and high filling pressures in the right chambers.
Day 3	Holter monitoring shows 8% of ventricular extrasystoles, and a beta-blocker is started.
Day 17	Patient underwent percutaneous occlusion of the arterial and venous termini of the fistula via the implantation of vascular plugs.
Day 19	Postprocedural echocardiogram is normal, and angiotomography reveals thrombus formation and a lack of residual flow through the fistula.
Day 19	Discharge from the hospital with the administration of anticoagulants.

ED = emergency department.

## Funding Support and Author Disclosures

The authors have reported that they have no relationships relevant to the contents of this paper to disclose.

## Appendix

Video 1 Three-Dimensional Reconstruction of the Fistula, Illustrating the Preoperative and Postoperative Features of the Patient's Anatomy, Along With the Devices Positioned Therein
